# The closing of the Species 2000 Organisation

**DOI:** 10.3897/BDJ.14.e209643

**Published:** 2026-08-24

**Authors:** Ralph Edward DeWalt, Christoph L. Haeuser, William Alexander Gray

**Affiliations:** 1 University of Illinois, Champaign, United States of America University of Illinois Champaign United States of America; 2 Museum fuer Naturkunde, Leibniz Institute for Evolution and Biodiversity Science, Berlin, Germany Museum fuer Naturkunde, Leibniz Institute for Evolution and Biodiversity Science Berlin Germany; 3 retired, Reading, United Kingdom retired Reading United Kingdom

**Keywords:** biodiversity informatics, Catalogue of Life, species names

## Abstract

This short communication is to inform the scientific community of the closing of Species 2000. The organisation was registered in the United Kingdom for 28 years and officially closed its doors as of 5 May 2026. It was responsible for building a "federation" of database organisations, taxonomists and sponsoring agencies that produced the most complete list of biological scientific names for life on Earth. Responsibility for continuing the Catalogue of Life data product and infrastructure has been transferred to the Catalogue of Life Foundation, a not-for-profit organisation registered in the Netherlands since May 2022. The former board members of Species 2000 wish the board of the Catalogue of Life Foundation best wishes for a fruitful continuance of the mission of the Catalogue of Life.

## Species 2000, its establishment and activities

The Species 2000 organisation started in 1996 with the goal of forming a “federation” of database organisations working closely with users, taxonomists and sponsoring agencies. The Species 2000 organisation, a limited company by guarantee, was established on 11 December 1997 with the United Kingdom Companies House (2026). The International Union of Biological Sciences was witness to its establishment. Species 2000 has been in continuous operation for over 28 years. The Catalogue of Life was its flagship activity with the aim of compiling a vetted list of species names for all life on Earth and to serve data to scientists, educators, government agencies, businesses and myriad other users. Data were aggregated from taxonomists, data scientists and government organisations. In 2001, the Catalogue of Life was first published online through a partnership with the Integrated Taxonomic Information System (ITIS 2026). In 2003, this partnership was formalised and a grant from the Global Biodiversity Information Facility (GBIF 2026) boosted the endeavour. The result is the most complete list of life on Earth with over 2 million accepted species names, literature references, millions of synonyms and a simplified classification served from its data product (COL 2026).

Under the leadership of Frank Bisby at the University of Reading beginning in 1997, Species 2000 thrived into the federation of taxonomic initiatives it aspired to since its origin. His sudden passing in October 2011 was a challenge for the organisation. Peter Schalk took over the leadership role. He managed to negotiate a stable home for the Catalogue of Life Executive Editor Yury Roskov. This position and a data manager were funded through the generosity of the David C. Eades Foundation that created an endowment for the Illinois Natural History Survey at the University of Illinois Urbana Champaign, USA. The endowment is still in operation today. The Species 2000 secretariat moved to Naturalis Biodiversity Center, Leiden, Netherlands in 2013. This arrangement ended amicably by mid-2023 and Species 2000 has been independent ever since.

## A new legal entity, the Catalogue of Life Foundation

Species 2000 has been registered in the United Kingdom Companies House throughout its existence. The United Kingdom leaving the European Union (EU) on 31 January 2020 limited the ability of Species 2000 to compete for funding within the EU. This change necessitated registration of a new legal entity in the EU to lead the Catalogue of Life. With some delays related to COVID, a new not-for-profit Catalogue of Life Foundation (COLF) was established in May 2022 in the Netherlands. From the Netherlands, the COLF could compete for funding to improve its data product, modernise its infrastructure and maintain its stated mission. Species 2000 Board members agreed that COLF would eventually take over all operations concerning the Catalogue of Life.

## Closing Species 2000

By late 2025, all outstanding funds from sponsors and debts to creditors had been resolved and the Board of Directors of Species 2000 began efforts to close the organisation. Financial documents were sent to accountants and submitted to the UK Companies House by October 2025 and bank accounts were closed. The Species 2000 membership was notified of the intent to close by early December and given two weeks to provide comment. The Board received only well-wishes from its members. With all requirements to close having been met, the United Kingdom Companies House (2026) notified the Board of full closure of Species 2000 on 5 May 2026.

All rights, responsibilities, data and infrastructure have been successfully transferred to the COLF (COL 2026). The former directors of Species 2000 (the authors) congratulate the new organisation for obtaining new funding, for creating an infrastructure capable of serving GBIF’s need for a new taxonomic backbone and for moving forward with robust, new infrastructure. The most recent annual release of data through the Catalogue of Life website contained nearly 2.5 million accepted species names (Bánki et al. 2026).
